# Selenium-Induced Toxicity Is Counteracted by Sulfur in Broccoli (*Brassica oleracea* L. var. *italica*)

**DOI:** 10.3389/fpls.2017.01425

**Published:** 2017-08-18

**Authors:** Ming Tian, Maixia Hui, Theodore W. Thannhauser, Siyi Pan, Li Li

**Affiliations:** ^1^Key Laboratory of Environment Correlative Dietology, Ministry of Education, College of Food Science and Technology, Huazhong Agricultural University Wuhan, China; ^2^Robert W. Holley Center for Agriculture and Health, United States Department of Agriculture – Agricultural Research Service, Cornell University, Ithaca NY, United States; ^3^College of Horticulture, Northwest A&F University Yangling, China; ^4^Plant Breeding and Genetics Section, School of Integrative Plant Science, Cornell University, Ithaca NY, United States

**Keywords:** broccoli, selenium, sulfur, Se toxicity, Se in protein, oxidative damage, antioxidant enzyme, gene expression

## Abstract

Selenium (Se) is an essential micronutrient for humans. Increasing Se content in food crops offers an effective approach to enhance the consumption of Se in human diets. A thoroughly understanding of the effects of Se on plant growth is important for Se biofortification in food crops. Given that Se is an analog of sulfur (S) and can be toxic to plants, its effect on plant growth is expected to be greatly affected by S nutrition. However, this remains to be further understood. Here, we evaluated the influence of Se treatments on broccoli (*Brassica oleracea* L. var. *italica*) growth when S was withheld from the growth nutrient solution. We found that Se was highly toxic to plants when S nutrition was poor. In contrast to Se treatments with adequate S nutrition that slightly reduced broccoli growth, the same concentration of Se treatments without S supplementation dramatically reduced plant sizes. Higher Se toxicity was observed with selenate than selenite under low S nutrition. We examined the bases underlying the toxicity. We discovered that the high Se toxicity in low S nutrition was specifically associated with an increased ratio of Se in proteins verse total Se level, enhanced generation of reactive oxygen species, elevated lipid peroxidation causing increased cell membrane damage, and reduced antioxidant enzyme activities. Se toxicity could be counteracted with increased supplementation of S, which is likely through decreasing non-specific integration of Se into proteins and altering the redox system. The present study provides information for better understanding of Se toxicity and shows that adequate S nutrition is important to prevent Se toxicity during biofortification of crops by Se fertilization.

## Introduction

Selenium (Se) is an essential micronutrient for humans and animals. Se deficiency affects a large number of the world population, which results from lack of Se in food crops growing on soils with low Se levels or phytoavailabilities (White and Broadley, 2009; White, 2016). Biofortification of food crops by means of Se fertilization provides an effective approach to increase the consumption of Se in human diets (Alfthan et al., 2015). Se biofortification in food crops also produces Se metabolites that serve as anticarcinogenic agents (Hatfield et al., 2014). Previous reports show that the monomethylated forms of Se, such as Se-methylselenocysteine and γ-glutamyl-Se-methylselenocysteine, have potent cancer chemopreventive activity (Ip et al., 2000; Dong et al., 2001). These anticarcinogenic compounds are enriched in the Se-biofortified *Brassica* crops (Lyi et al., 2005; Ramos et al., 2011b; Ávila et al., 2013, 2014). Thus, a better understanding of Se metabolism is important for Se biofortification and enrichment in food crops.

Sulfur is an essential nutrient for plants and plays diverse functions in plants, such as acting in the redox system to protect the cells from oxidative stress damage (Takahashi et al., 2011). Both S and Se form part of the VIA chalcogen group of elements and have the similar physical and chemical property. As an S analog, Se is believed to use S uptake and assimilation pathways in plants (Sors et al., 2005; White, 2016; Gupta and Gupta, 2017; Schiavon and Pilon-Smits, 2017). Selenate (SeO_4_^2-^) and selenite (SeO_3_^2-^) are the main forms of Se in soils with selenate more soluble in alkaline soils and selenite more soluble in acidic soils. Plants take up selenate from soils using the root sulfate transporters (Terry et al., 2000). The transporter selectivity for Se and S depends on the plant species and the status of S (White et al., 2004). Selenate and sulfate compete for uptake by the transporters, but stimulate each other’s uptake at low concentrations (Lyi et al., 2005; Boldrin et al., 2016). The uptake of selenite in plants was originally believed to be passive through diffusion, but more recent studies reveal active transport that partially involves phosphate transporters (Li et al., 2008; Zhang et al., 2014). Following the uptake, selenite is readily converted into organic Se compounds in roots, whereas selenate is rapidly translocated to shoots and either stored or assimilated in plastids via the sulfur metabolic pathway (White, 2016; Gupta and Gupta, 2017; Schiavon and Pilon-Smits, 2017).

Selenium is both essential and toxic for most life forms. While there is no evidence to suggest that Se is essential to higher plants, Se at low doses is generally beneficial to plant growth and development (Gupta and Gupta, 2017). However, when the optimum concentrations of Se are exceeded, Se becomes toxic to plants. The toxicity is believed to be caused either by non-specific integration of seleno-amino acids into proteins to disrupt the protein normal functions or by generation of reactive oxygen species (ROS) to induce oxidative stress in plants (Gupta and Gupta, 2017). The extent of toxicity was suggested to be associated with the Se/S ratio in plant tissues rather than the Se content *per se* (White et al., 2004; Kassis et al., 2007). Given that Se is an analog of S and shares S metabolism pathways, the effect of Se on plant growth is expected to be greatly affected by S nutrition. However, this remains to be further understood.

Broccoli (*Brassica oleracea* L. var. *italica*) contains multiple nutrients (i.e., vitamins and minerals) and many health beneficial compounds, especially sulfur-containing metabolites (Vasanthi et al., 2009; Tian et al., 2016a). As an S and/or Se secondary accumulator, broccoli is able to accumulate high levels of S and Se when grown in S and Se rich environments. Previous studies in broccoli and other plants examine the interactions of Se and S nutrition in the presence of adequate amounts of S (White et al., 2004; Lyi et al., 2005; Boldrin et al., 2016; Tian et al., 2016b). In this study, we compared the effect of Se treatments on plant growth when S was present and absent from the growth nutrient solution. Much enhanced Se toxicity to plant growth was observed when S level was low. We investigated the basis underlying Se toxicity or protection by S. Our results reveal that Se treatments increase the ratio of Se in proteins vs total Se level and enhance lipid peroxidation causing increased cell membrane damage. S counteracts Se toxicity, which is likely through altering the redox system. This study provides data showing that adequate S nutrition is important to prevent Se toxicity when biofortification of crops by Se fertilization.

## Materials and Methods

### Plant Materials and Experimental Designs

Broccoli seeds of two commercial varieties (DIPLOMAT and GYPSY) were obtained from Harris Seeds (Rochester, NY, United States) and used in this study. Seeds were germinated in roll sheets of moistened filter papers for 5 days in a growth chamber at 25°C with 16-h light/8-h dark. The young seedlings were then transferred into a container with Hoagland solution (Hoagland and Arnon, 1938) and grown in a greenhouse. After conditioning for 2 days in the full nutrient solution, uniform seedlings were transferred into 2.2 L black pots containing the Hoagland solution with various treatments and grown in the same greenhouse at 23–25°C with a 14-h light and 10-h dark photoperiod under constant aeration.

Treatments with and without S (Na_2_SO_4_) containing increased levels of selenate (Na_2_SeO_4_) or selenite (Na_2_SeO_3_) were divided into 10 groups (**Table 1**). The nutrient solutions were changed twice every week. After 2 weeks of treatments, 80 plants (2 varieties × 10 treatments × 4 biological repeats) were harvested individually and separated into shoots and roots. Some of the fresh leaves were used directly for membrane damage test. The others and roots were either dried for mineral analysis or immediately frozen in liquid nitrogen and stored at -80°C for RNA extraction and enzyme activity analysis.

**Table 1:** Various Se and S treatments used in this study.

	A	B	C	D	E	a	b	c	d	e
Na_2_SO_4_ (mM)	1	1	1	1	1	0	0	0	0	0
Na_2_SeO_3_ (μM)	0	20	0	40	0	0	20	0	40	0
Na_2_SeO_4_ (μM)	0	0	20	0	40	0	0	20	0	40

### Total Se and S Level Analysis

The ionomics of broccoli leaf and root tissues containing a total 26 elements including Se and S were determined using an inductively coupled plasma (ICP) trace analyzer emission spectrometer (model ICAP 61E trace analyzer, Thermo Electron, San Jose, CA, United States) essentially as described previously (Figueiredo et al., 2017). Briefly, the dried tissues (approximately 100 mg) were weighed into borosilicate glass tubes, acid-digested in 2.0 mL of H_3_NO_3_ with 2.0 mL of HClO_4_ at 120°C for 1 h, and then at 220°C until HClO_4_ fumes were observed. The digested samples were solubilized with 20 mL of 18 MΩ water before analysis. Four biological replicates were analyzed.

### Analysis of Se Levels in Proteins

Selenium in proteins was extracted and analyzed following the method as described by Wu et al. (2014). Frozen fresh tissues (0.1 g) were ground into powder and extracted using 0.5 mL of cold (-20°C) trichloroacetic acid (TCA)/acetone (0.1 g mL^-1^). The homogenized samples were centrifuged at 12,000 *g* for 5 min at 4°C to collect the precipitated proteins. The supernatant was discarded and the pellets were resuspended in 0.1 mL of cold TCA/acetone (-20°C) followed by centrifugation again. This step was repeated one more time. The final pellets were resuspended in 1.5 mL 1% SDS, and incubated between 60 and 70°C for 1–2 h. Se levels in proteins were analyzed by ICP. The experiment was repeated with four biological replicates for all samples.

### *In Situ* ROS Detection

ROS generated in leaves of broccoli were detected as described previously (Zhou et al., 2009). Briefly, fresh young leaves from multiple biological repeats were vacuum infiltrated in 0.1% nitro-blue tetrazolium (NBT) in 10 mM KPO_4_ buffer (pH 7.8). The samples were incubated in the NBT staining solution at room temperature overnight. Stained leaves were treated in boiling ethanol (95%) for 15 min to remove chlorophylls prior to photography.

### Lipid Peroxidation Analysis

Lipid peroxidation in the leaves was estimated by measuring the level of malondialdehyde (MDA, a production of lipid peroxidation) following the method of Dhindsa et al. (1981) with minor modification. Leaf samples (50 mg) were homogenized in 1 mL of 0.1% TCA, followed by centrifugation at 10,000 *g* for 5 min. To 0.2 mL aliquot of the supernatant, 0.8 mL of 20% TCA containing 0.5% thiobarbituric acid (TBA) was added. The mixture was heated at 95°C for 30 min, quickly cooled on ice-bath, and centrifuged at 10,000 *g* for 10 min. The absorbance of the supernatant was measured at 532 nm using a spectrometer and the value at 600 nm for non-specific absorption was subtracted. The concentration of MDA was calculated using the extinction coefficient of 155 mM^-1^ cm^-1^. The experiment was repeated with four biological replicates for all samples.

### Determination of Membrane Permeability

Membrane permeability represented by the percentage of electrolyte leakage was measured by electrolyte conductivity (EC) according to the method as described (Yan et al., 1996) with minor modification. The fresh leaves were cut into 1 cm^2^ pieces and placed in a beaker containing 10 mL deionized water. The beaker was then placed at 30°C water bath for 2 h. The conductivity of solution was measured by EC tester to record *C*_1_. After boiling for 2 min, the conductivity of solution was measured again and recorded *C*_2_. The percentage of electrolyte leakage was calculated following the equation: percent EC = *C*_1_/*C*_2_ × 100. The experiment was repeated with four biological replicates for all samples.

### Analysis of Antioxidant Enzyme Activities

Ascorbate peroxidase (APX, EC 1.1.11.1) and catalase (CAT, EC 1.11.1.6) activities were measured as described previously (Ramos et al., 2011a). For these assays, 200 mg powdered leaves were extracted in 1.5 mL ice-cold extraction buffer containing 50 mM KH_2_PO_4_–KOH (pH 7.5), 0.1 mM ethylenediaminetetraacetic acid (EDTA), 0.3% (w/v) Triton X-100, and 4% (w/v) insoluble polyvinylpolypyrrolidone. The mixture was kept on ice for 10 min, followed by centrifugation at 12,000 *g* for 10 min at 4°C. The supernatant was used immediately for enzyme activity assays.

To detect APX activity, the assay mixture (1 mL) contained 50 mM HEPES–KOH (pH 7.6), 0.1 mM EDTA, 0.2 mM H_2_O_2_, 0.5 mM reduced ascorbate (AsA), and enzyme extract. The reaction was initiated by adding H_2_O_2_, and the decrease in absorbance at 290 nm was recorded. The enzyme activity was calculated using the extinction coefficient of 2.8 mM^-1^ cm^-1^.

To detect CAT activity, the reaction mixture (1 mL) contained 100 mM potassium phosphate buffer (pH 7.0), 10 mL 10% (w/v) H_2_O_2_ and enzyme extract. The reaction was initiated by adding H_2_O_2_ and the decrease in absorbance at 240 nm was measured. The enzyme activity was calculated using the extinction coefficient of 39.4 mM^-1^ cm^-1^. All experiments were performed with four biological replicates.

### RNA Extraction and Quantitative PCR Analysis

Total RNA from 0.1 g of leaves or roots of broccoli was extracted using TRIzol according to the manufacturer’s instructions (Life Technologies) and reverse-transcribed into cDNA using Superscript III Reverse Transcriptase (Invitrogen). A quantitative reverse transcription polymerase chain reaction (qRT-PCR) was performed using the SYBR Green Universal Master Mix (PE Applied Biosystems) in an ABI750 Real-Time PCR system as described previously (Lyi et al., 2007; Zhou et al., 2011). The gene-specific primers used are listed in **Table 2**. Three biological repeats and two technical repeats were carried out for each treated sample.

**Table 2:** List of primers used in this study.

Genes	Forward primer (5′–3′, top) Reverse primer (5′–3′, bottom)	PCR size (bp)	GenBank accession
*BoActin*	CCGAGAGAGGTTACATGTTCACCAC	376	XM_013763767.1
	GCTGTGATCTCTTTGCTCATACGGTC		
*BoAPS1*	AGACGACGAGCAAAAGGCTA	145	XM_013774858.1
	GGTTGTACCCCATGTTCTGG		
*BoAPS2*	CGTTGACACTCCCATCACTG	202	XM_013749911.1
	TTGATCGGAGAAAGAGGATG		
*BoAPS3*	TGAAACAGCACGAGAAGGTG	197	XM_013752442.1
	ACGTTTCTCCACAGGGTGAC		
*BoAPR1*	TTGCTAAGAAGCTAGAGAAT	140	XM_013757845.1
	TGGTCTCCCAGTTAAATGAG		
*BoAPR2*	TCTTTGGTTACCCGTGCTTC	107	XM_013781409.1
	GGAGAAGCCTCTTCCAGCTT		
*BoAPR3*	TTCCCTTCCTCAGAGCTCAA	149	XM_013747679.1
	TCCTTTGCAACTGACTGCAC		
*BoSultr1;1*	GATTCTGCTGCAAGTGACGA	126	XM_013763767
	ACGCGAATGATCAAGATTCC		
*BoSultr1;2*	AAGCAGTTCATGCTCGGTCT	149	XM_013762968.1
	AGCGAGCTTAGCGTATCCAA		

### Statistical Analysis

The significant difference among treatments was determined using Duncan’s multiple-range test (*P* < 0.05 as difference; Duncan, 1955). All data are shown as the means ± SE of at least three biological replicates.

## Results

### Effect of Se Treatments on Plant Growth in the Presence and Absence of S Supplementation

**FIGURE 1:**
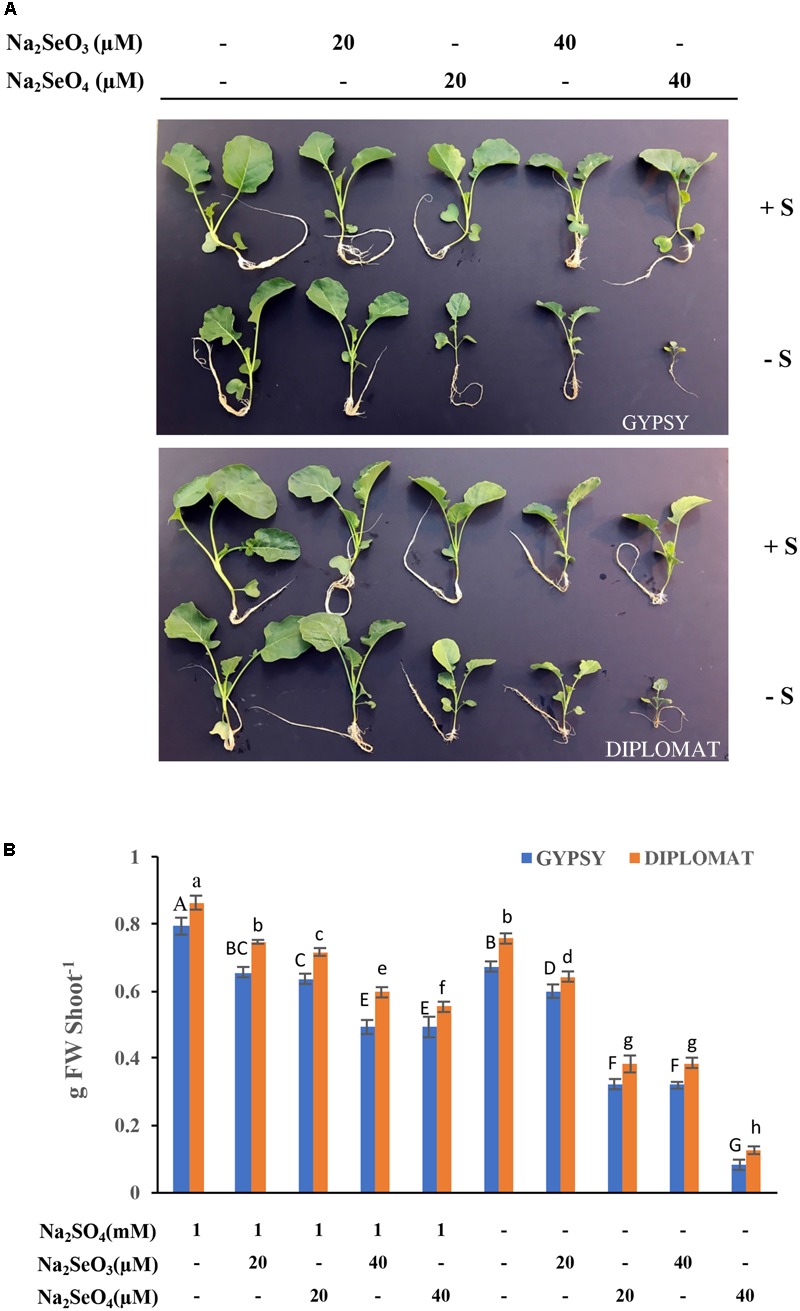
Broccoli plant growth in response to different Se treatments in the presence and absence of S supplementation. **(A)** Representative images of plant growth phenotype from four biological replicates. Two cultivars were used in the study and the plants were treated for 2 weeks. **(B)** The corresponding shoot biomass of these two broccoli cultivars subjected to different treatments for 2 weeks. Data represents means from four independent plants. Error bars indicate ±SE. Different letters above the columns for each cultivar indicate significant differences at *p* < 0.05 by Duncan’s multiple range test. FW, fresh weight.

Selenium can be toxic to plants. Its toxicity is expected to be effected by S due to the fact that Se and S are analogs. To examine the toxicity of Se to plant growth and the role of S in protecting plants from Se toxicity, two broccoli cultivars were grown in Hoagland nutrient solution and treated with increased amounts of Na_2_SeO_4_ or Na_2_SeO_3_ in the presence or absence of 1 mM Na_2_SO_4_ supplementation for 2 weeks (see **Table 1**). As shown in **Figure 1A**, the plant growth phenotypes varied in response to Se treatments. In the presence of S, the plant sizes were gradually reduced with the increased additions of selenate and selenite to 20 and 40 μM in comparison with the non-Se treated controls for both cultivars. No dramatic differences in plant growth phenotypes were observed when plants were treated with selenate and selenite at the dosages used (**Figure 1**). However, when S was withdrawn from the growth solution, high levels of Se treatments at 40 μM dramatically reduced plant sizes with much smaller plants (**Figure 1A**). Selenate in general is less toxic than selenite to plants (Terry et al., 2000). Interestingly, high Se toxicity with much smaller plant sizes was observed with the selenate than the selenite treated plants when exposed to the same concentrations of Se in the absence of S supplementation (**Figure 1A**).

**FIGURE 2:**
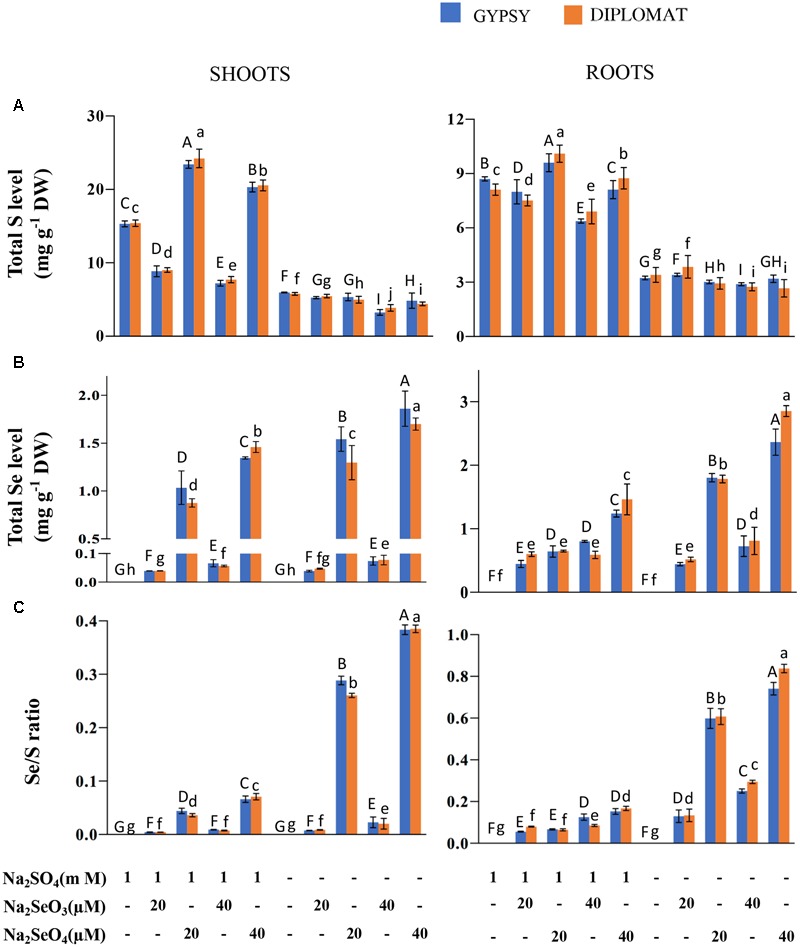
Total S and Se levels and Se/S ratio in two cultivars of broccoli subjected to different forms and levels of Se treatment with and without S supplementation. **(A)** Total S levels in shoots and roots of plants treated for 2 weeks. **(B)** Total Se levels in shoots and roots of plants treated for 2 weeks. **(C)** Se/S ratios that were calculated with total Se levels divided by total S levels. Data represents means from four independent biological replicates. Error bars indicate ±SE. Different letters above the columns indicate significant differences at *p* < 0.05 by Duncan’s multiple range test.

The fresh weights of these plants were measured. Consistently with plant growth phenotypes, addition of 20 and 40 μM Na_2_SeO_4_ or Na_2_SeO_3_ in the presence of S gradually, but significantly, reduced plant fresh weights in comparison with non-Se treated controls for both cultivars (**Figure 2B**). No dramatic differences in plant fresh weights were observed between selenate and selenite treatments at the Se dosages used. However, when S was withdrawn from the nutrient solution, the plant biomass was greatly affected by 20 μM of selenate in comparison with selenite. At 40 μM of Se, selenate treatment significantly inhibited plant growth to produce plants that retained only one-fourth of the biomass in comparison with selenite treatment (**Figure 2B**). The selenite treatment at 40 μM showed similar biomass as selenate at 20 μM (**Figure 2B**). These results indicate that selenate is more toxicity than selenite when S nutrition was low and S can protect plants from Se toxicity.

### Selenium and S Accumulation in Response to Se and S Treatments

As a Se/S accumulator, broccoli is known to accumulate significant amounts of these elements (Lyi et al., 2005; Ramos et al., 2011b; Ávila et al., 2013). We first examined the total S and Se levels in broccoli shoots and roots treated with different forms of Se in the presence and absence of S in the nutrition solution. In the presence of 1 mM Na_2_SO_4_ in the growth nutrient solution, selenite treatments at 20 and 40 μM significantly reduced the total S levels in shoots of both broccoli cultivars in comparison with the control (**Figure 2A**). However, selenate treatments significantly enhanced the total S content in shoots as observed previously in wheat (Boldrin et al., 2016). Over 1.6-fold increase in total S content was observed with the addition of 20 μM Na_2_SeO_4_. In the absence of S supplementation in the nutrition solution, the total S levels in shoots were low and showed no stimulated enhancement by selenate (**Figure 2A**). The low amounts of S found were likely resulted from retaining of S during germination prior treatments. In contrast to shoots, Se treatments generally exhibited less effect on total S levels in the roots although significant differences among treatments were observed. While selenite treatment at 40 μM slightly reduced S accumulation in the presence of S, selenate treatments at both 20 and 40 μM increased total S levels in roots. When S was withdrawn from the nutrition solution for 2 weeks, the total S accumulation among various treatments did not change dramatically in roots (**Figure 2A**).

Broccoli responded positively to Se supplementation. The total Se levels in both shoots and roots increased with increased dosages of Se (**Figure 2B**). Much higher levels of Se were observed when treated with selenate than selenite (**Figure 2B**). The difference is likely due to efficient translocation of selenate to shoots but less effective transport for selenite. At the same doses of selenate treatments, up to twofold more Se was accumulated in shoots and roots when plants were grown in the nutrient solution without S than with S, showing increased uptake and accumulation of Se when S was absent in the solution (**Figure 2B**).

It is well known that uptake, accumulation, and utilization of elements in plants can be affected by other elements. In particular, it has been reported a significant relationship between S and Fe, in which S deficiency impairs Fe acquisition and accumulation and vice versa Fe deficiency affects S uptake and accumulation (Ciaffi et al., 2013; Paolacci et al., 2014). Thus, in addition to S and Se, we also examined the total contents of other elements by ICP. As shown in Supplemental Figure S1, the various Se and S treatments exerted minimal effects on the total levels of other elements such as P, K, Na, Ca, and Mg, as well as Fe, Zn, Cu, Al, and Mn in both shoots and roots of these two cultivars under the treatment conditions. The results were different from that observed in durum wheat, which shows a decrease of Fe concentration under S deficiency (Ciaffi et al., 2013). The discrepancy could result from species difference. Indeed, our previous study in examining the effect of Se treatments on the mineral accumulation in broccoli germplasm reveals diverse genetic variation even within the same species in response to Se treatment (Ramos et al., 2011b).

The Se/S ratio in plant tissues was suggested to affect the extent of Se toxicity (White et al., 2004; Kassis et al., 2007). The Se/S ratio for the same Se treatment was much higher in the absence than in the presence of 1 mM Na_2_SO_4_ supplementation in the nutrient solution (**Figure 2C**). The highest Se/S ratio was observed with 40 μM Na_2_SeO_4_ in the minus S treatment (**Figure 2C**), consistent with the high Se toxicity phenotype (**Figure 1**). Interestingly, while the growth inhibition at 20 μM of selenate and 40 μM of selenite was not statistically different when grown in the absence of S supplementation (**Figure 1**), the Se/S ratio was significantly lower for the selenite than selenate treatment for both shoots and roots (**Figure 2C**), indicating that the Se form is also important in addition to the Se/S ratio for the extent of Se toxicity.

**FIGURE 3:**
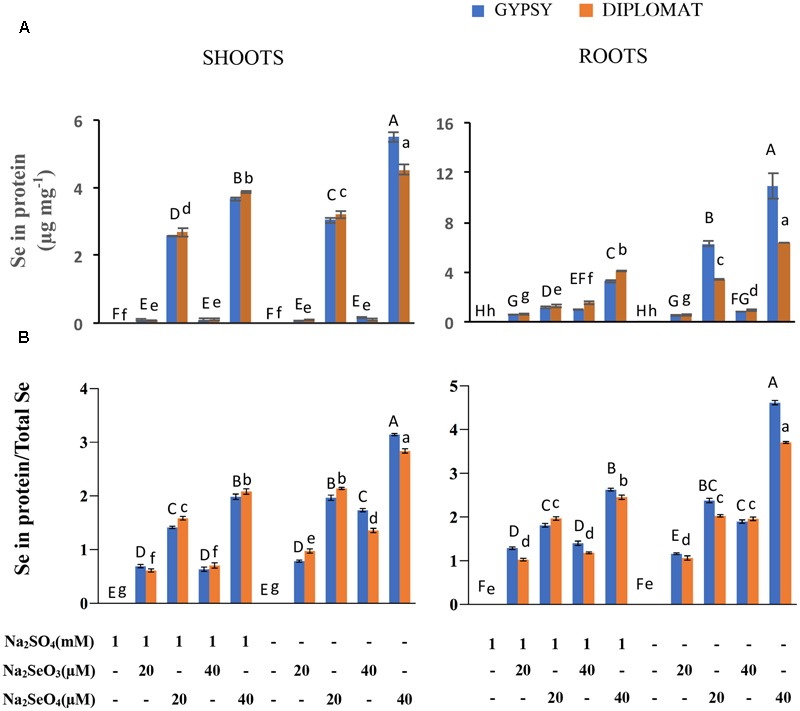
Effect of different Se/S treatments on the levels of Se in proteins and the relative ratios of Se in proteins vs total Se content. **(A)** Total Se levels in proteins in shoots and roots of plants treated for 2 weeks. **(B)** Relative ratios of Se in proteins/total Se levels. Data represents means from four independent biological replicates. Error bars indicate ±SE. Different letters above the columns indicate significant differences at *p* < 0.05 by Duncan’s multiple range test.

The non-specific integration of the selenoamino acid selenocysteine (SeCys) and selenomethionine (SeMet) into proteins is believed to be the major contributor of Se toxicity in plants (Sors et al., 2005). To examine whether the increased Se toxicity was associated with enhanced Se accumulation in proteins, the total Se in protein extracts was measured. Under the same concentrations of selenate treatments, significant more Se accumulated in proteins from plants growing without S addition in the nutrient solution than with S, particularly in the roots where up to threefold more Se was found (**Figure 3A**). These results support the inhibitory effect of non-specific integration of selenoamino acids to plant growth.

The relative ratios between Se in proteins and total Se content were also examined. In the presence of S treatment, significantly increased ratios were observed with increased levels of selenate treatments in both shoots and roots, whereas the ratios were not significantly different with selenite treatments (**Figure 3B**). Noticeably in the absence of S supplementation, the ratios of Se content in proteins/total Se between 20 μM of selenate and 40 μM of selenite were similar without significant difference in roots. The highest ratio was found in 40 μM of selenate treatment, which produced the smallest size of plant (**Figure 1**). These results suggest a direct association between the ratio of Se in proteins/total Se and the extent of Se toxicity.

### Effect of Se Treatments on ROS Production, Lipid Peroxidation, and Membrane Permeability

**FIGURE 4:**
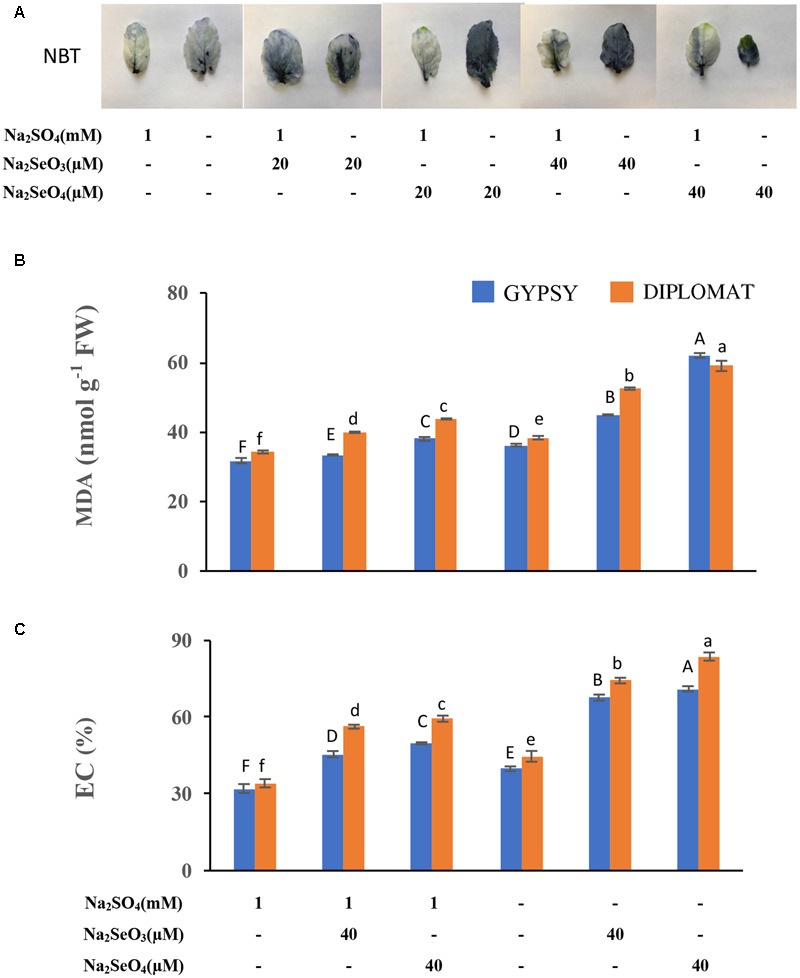
Analysis of ROS production, lipid peroxidation, and membrane damage in leaves following various Se/S treatment for 2 weeks. **(A)** Representative leaf images from four independent plants following NBT staining. The purple precipitations indicate the production of ROS. **(B)** Level of lipid peroxidation as indicated by the production of malondialdehyde (MDA). **(C)** Extents of membrane damage as indicated by electrolyte conductivity (EC) values. Data represents means from four biological replicates. Error bars indicate ±SE. Different letters above the columns indicate significant differences at *p* < 0.05 by Duncan’s multiple range test.

To further examine the basis underlying Se induced growth inhibition when S level was low, we first examined ROS production. Selenium as other stress factors has been reported to induce ROS production in plants (Zhou et al., 2009; Dietz and Sunkar, 2010). NBT stain is commonly used to detect ROS production. Increased NBT staining was observed in leaves after treated with both selenite and selenate (**Figure 4A**). Much darker color was observed in leaves treated with same Se concentration in the absence than the presence of S in the growth solution, indicating an increased ROS production.

Prolonged maintenance of ROS in tissues often leads to lipid peroxidation and membrane damage (Dietz and Sunkar, 2010). To further examine the toxic effect of Se treatments, lipid peroxidation with the production of MDA was examined to associate with the growth inhibition of Se in the presence and absence of S supplementation in the growth solution. As shown in **Figure 4B**, supplementation of high concentrations of Se produced significantly more MDA. Significantly enhanced production of MDA was observed when Se treated plants grew in the nutrient solution without S than with S supplementation. More MDA production was found when plants were treated with selenate than selenite (**Figure 4B**).

We further examined membrane permeability in plants treated with Se in the presence and absence of S in the nutrient solution. Se treatments resulted in increased EC, but significantly higher EC values were observed when plants were grew in the nutrient solution without S (**Figure 4C**), indicating an enhanced damage of cell membranes.

### Effect of Se Treatment on the Activities of Antioxidative Enzymes

**FIGURE 5:**
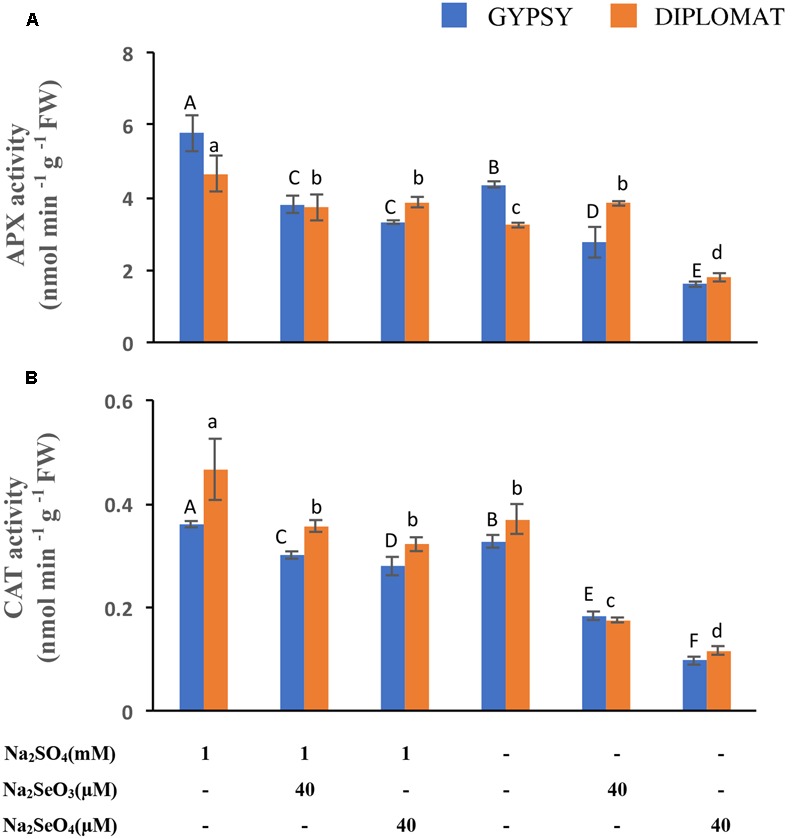
Ascorbate peroxidase (APX) and catalase (CAT) activities in leaves following various Se/S treatment for 2 weeks. **(A)** Activity of APX. **(B)** Activity of CAT. Data represents means from four biological replicates. Error bars indicate ±SE. Different letters above the columns indicate significant differences at *p* < 0.05 by Duncan’s multiple range test.

APX and CAT are part of the antioxidative system to protect cells against the oxidative damage. Their activities following Se treatment were examined. APX and CAT enzyme activities were reduced under both selenate and selenite treatments at 40 μM (**Figures 5A,B**). However, dramatic reduction of both enzyme activities was observed when the plants were exposed to 40 μM of selenate in the nutrient solution without S supplementation (**Figures 5A,B**). The activities of APX and CAT exhibited the same trend of change as plant growth in response to the different Se and S treatments (**Figure 1**), indicating an important role of these antioxidative enzymes in protecting plants from Se induced toxicity. The results also suggest that these enzyme activities had a negative correlation to the oxidative damage observed (**Figure 4**), and S could increase the enzyme activities to protect plants from Se toxicity.

### Expression of Genes Involved in S/Se Transport and Assimilation

**FIGURE 6:**
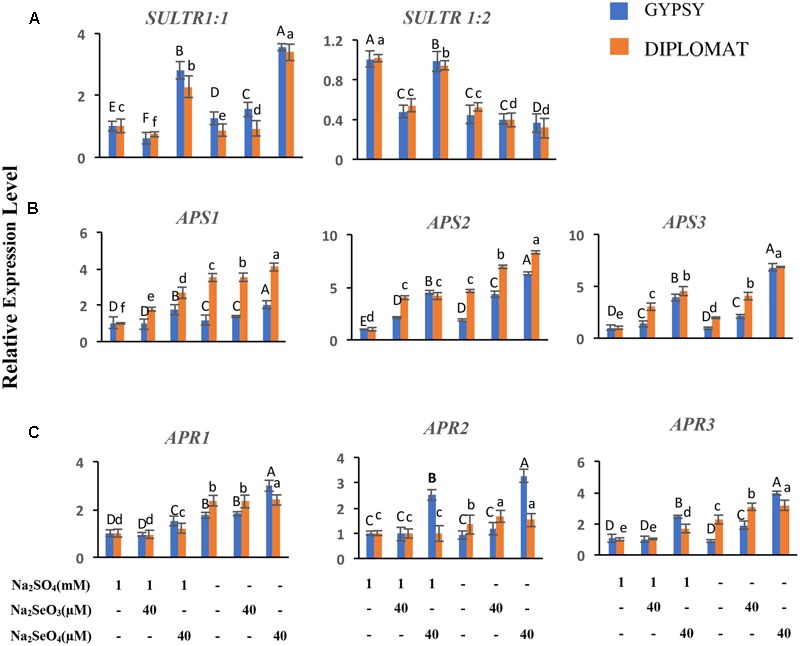
Relative expression of genes involved in S/Se transport and assimilation in broccoli following various Se/S treatments for 2 weeks. **(A)** Transcript levels of S transporters in roots. **(B,C)** Transcript levels of *APS* and *APR* genes in leaves. The expression levels were measured by qRT-PCR. The expression of genes in non-treated samples with 1 mM of sulfate supplementation was set to 1. Data are means of three biological replicates with two technical trials each. Error bars indicate ±SE. Different letters above the columns indicate significantly different according to Duncan’s multiple range test at *p* < 0.05.

Plants take up and metabolize Se via the S transporters and assimilation pathways (White, 2016; Schiavon and Pilon-Smits, 2017; Gupta and Gupta, 2017). The effects of Se treatments in the presence and absence of S in the nutrient solution on the expression of S transporters in roots and S assimilation pathway genes in shoots were examined. *Sultr 1;1* and *Sultr 1;2* represent high-affinity S transporters expressed in roots for uptake of sulfate from the rhizosphere (Saito, 2004). While selenite exposures exhibited limited effect on *Sultr 1;1* expression, selenate supplementation at 40 μM significantly upregulated *Sultr 1;1* transcript levels with up to 2.5-fold increase under both with and without S treatments (**Figure 6A**). *Sultr 1;2* showed different pattern of expression. Its transcript level was reduced when S was withdrawn from the nutrient solution. In the presence of S supplementation, selenite significantly decreased *Sultr 1;2* expression to half, while selenate showed relative small effect on its expression (**Figure 6A**). The increased expression of *Sultr 1;1* in the selenate treated plants showed an association with Se accumulation, suggesting an important role of *Sultr 1;1* in selenate uptake here.

ATP-sulfurylase (APS) and APS reductase (APR) are two key enzymes in S/Se assimilation pathway (Saito, 2004). Their family gene expressions related to Se accumulation in leaves were also examined. Significantly increased expressions of *APS2* and *APS3* were observed in response to Se treatments and when plants were treated with selenate in S minus solution for both cultivars (**Figure 6B**). Similarly, significantly increased expression of *APR1* and *APR3* were also noted when plants were treated with selenate in S minus solution than in the presence of S (**Figure 6C**). Higher transcript level of *APS3* was noted in selenate-treated plants in S minus solution, suggesting an enhanced assimilation, which is consistent with high level of Se in proteins (**Figure 3A**).

## Discussion

Selenium fertilization to biofortify crops provides an effective approach not only to combat Se deficiency but also to enhance chemopreventive compounds (Zhu et al., 2009; Malagoli et al., 2015). However, Se treatments can result in toxicity to crops (Gupta and Gupta, 2017). Broccoli is rich with health-beneficial compounds and has high capacity to accumulate Se. We examined Se toxicity in broccoli and determined how Se toxicity is affected by S nutrition by treating two broccoli varieties with Se in the presence and absence of S supplementation in the growth solution. The study reveals that Se was highly toxic to plants when S nutrition was poor. The toxicity was found to be associated with a high ratio of Se in proteins/total Se and an increase in oxidative damage. Increase of S supplementation counteracted Se toxicity, which was likely through reducing the non-specific integration of Se into proteins and mediating the redox enzyme activities.

While broccoli as a Se accumulator has relatively high capacity to tolerate Se, Se treatments especially selenate exposure at 40 μM dramatically inhibited plant growth when S was withdrawn from the nutrient solution (**Figure 1**). In comparison, the same Se treatments showed much less growth inhibition when S was supplied, indicating the protective role of S in reducing Se toxicity in plants. Se toxicity was suggested to be directly associated with Se/S ratio (White, 2016). We first determined the S and Se levels in the treated plants. The total S accumulation in shoots was affected by the forms of Se. Selenate treatments at the dosages used significantly enhanced shoot S level, while selenite decreased it, consistent with previous studies in broccoli and other crops (White et al., 2004; Ramos et al., 2011b). The selenate-induced S accumulation was shown to be due to selenate treatment mimicking S deficiency in activating specific sulfate transporters to stimulate S uptake (Boldrin et al., 2016).

Similarly, total Se contents in shoots and roots were also affected by the forms of Se supplied. While selenite treatments resulted in low Se accumulation in shoots, increased concentrations of selenate exposure led to enhanced Se levels in both shoots and roots. Previous reports also show that selenate is more effective than selenite in promoting Se accumulation in different crops (Chen et al., 2002; Sharma et al., 2010; Ramos et al., 2011a; Ávila et al., 2013). Much high levels of Se accumulation were observed when S was eliminated from the nutrient solution containing selenate (**Figure 2B**). The high Se accumulation in the selenate treated shoots and roots likely resulted from less competition for sulfate transporters in the absence of sulfate in the growth solution. As a result, the Se/S ratio was severalfold higher in both shoots and roots when broccoli was treated with the same doses of selenate with and without addition of S in the growth solution (**Figure 2C**). A reverse linear relationship between Arabidopsis growth and its shoot Se/S concentration ratio was reported (White et al., 2004). The similar growth inhibition by 20 μM selenate and 40 μM selenite but with quite different total Se/S ratio suggests that factors other than the total ratios also affect Se toxicity.

Non-specific integration of seleno-amino acids SeCys and SeMet instead of cysteine and methionine into proteins is thought to be a main cause for Se induced toxicity in many plants (Terry et al., 2000; Sors et al., 2005; Broadley et al., 2006). Determination of Se content in proteins revealed similar pattern of changes as total Se with different forms of Se treatments in the presence and absence of S supplementation (**Figure 3A**). Indeed, accumulation of organic Se was reported to be proportional to total Se levels in broccoli and other plants (Zayed et al., 1998; Ramos et al., 2011b; Banuelos et al., 2015). Comparison of the ratios of Se in proteins vs total Se revealed that the large growth inhibition in the 40 μM of selenate-treated plants without S supplementation was associated with much high ratio (**Figure 3B**). Moreover, the similar growth inhibitions by 20 μM selenate and 40 μM selenite were correlated with statistically indifferent ratios of Se in proteins vs total Se. These results indicate an association of the ratio of Se in proteins/total Se with Se toxicity. The data provides information to explain the high toxicity of selenate compared to selenite to plant growth in the absence of S supplementation.

Selenium can be directly toxic to plants, which is believed to be caused by the generation of ROS to induce oxidative stress in plants (Zwolak and Zaporowska, 2012; Gupta and Gupta, 2017). Indeed, Se treatments have been shown to induce the formation of ROS as assessed by NBT staining in a number of plant species (Ellis et al., 2004; Tamaoki et al., 2008; Lyons et al., 2009; Zhou et al., 2009; Golam et al., 2017). Consistent with those studies, we observed ROS production especially when S was withdrawn from the Se-treated growth solution. S addition clearly reduced ROS production (**Figure 4A**). The prolonged maintenance of ROS such as O_2_^-^ and H_2_O_2_ in tissues can lead to lipid peroxidation and damage the cell membranes (Dietz and Sunkar, 2010). Accordingly, the ROS production was found to be associated with increased lipid peroxidation and membrane permeability in broccoli plants. Broccoli leaves treated with Se in the absence of S supplementation generated more MDA and led to cell membrane damage with increased ion leakage, while the leaves of plants grown at the same level of Se but with the addition of S generated less MDA and ion leakage. The reduced growth in plants treated with 40 μM of selenate in the absence of S supplementation was clearly associated with high ROS induced damages. Induction of ROS production under flooding has been also shown to result in induced lipid peroxidation and membrane damage in corn leaves (Yan et al., 1996).

To cope with the toxic effect of ROS, plants form an antioxidative system to protect cells against the oxidative damage. Antioxidant enzymes such as APX and CAT are part of the antioxidative system. Selenium treatments have been shown to affect the antioxidant enzyme activities which in turn influence plant growth (Hartikainen et al., 2000; Ramos et al., 2011a). Here we showed that the growth inhibition of plants treated with Se in the absence of S supplementation was directly correlated with the reduction of APX and CAT enzyme activities. Dramatically low APX and CAT enzyme activities were detected in plants that exhibited worse growth phenotype when treated with selenate without S supplementation (**Figures 1, 5**). The enzyme activities of APX and CAT, however, were reduced to a much less extent when plants treated with both S and Se, consistent with slightly reduced plant growth, showing a functional role of S in protecting plants against Se toxicity. A similar work showed that sulfur could ameliorate arsenic toxicity by enhancing APX and CAT enzyme activities in rice (Dixit et al., 2015).

*Sultr 1;1* and *Sultr 1;2* are two high-affinity sulfate transporters that play the major role for the uptake of S and Se in roots. Previous studies reveal that selenate treatment can mimic S deficiency to activate *Sultr 1;1* expression, leading to enhance both Se and S uptake (Rouached et al., 2008; Boldrin et al., 2016). Consistent with this, a significantly increased *Sultr 1;1* transcription was detected when the broccoli plants were treated with selenate (**Figure 5A**), correlating with an increased Se levels in shoots.

## Conclusion

In conclusion, our data shows that Se treatments could be highly toxic to plants with the increased integration of Se into proteins and the generation of ROS to cause cell membrane damage, particularly in low S nutrition. The Se toxicity could be greatly reduced with adequate amounts of S. The present study provides information for better understanding of Se toxicity and the interaction between Se and S. It offers guidance for Se biofortification to enhance Se content in crops without negative effects on plant growth.

## Author Contributions

MT, SP, LL designed experiments. MT performed experiments. MT, MH, TT, SP, and LL analyzed and interpreted data. MT and LL wrote the manuscript with contributions of all the authors.

## Conflict of Interest Statement

The authors declare that the research was conducted in the absence of any commercial or financial relationships that could be construed as a potential conflict of interest.
