# Supplementary material for: Selenium-Induced Toxicity Is Counteracted by Sulfur in Broccoli (Brassica oleracea L. var. italica)
Source: Front Plant Sci. 2017 Aug 18;8:1425. doi: 10.3389/fpls.2017.01425 (PMC5563375; doi:10.3389/fpls.2017.01425)
Supplement: Supplementary file 1 [file Presentation_1.PDF]

SUPPLEMENTAL FIGURE S1

Ionomic analysis of total mineral levels in two cultivars of broccoli subjected to different forms and levels of Se treatment with and without S supplementation. Total 26 elements were analyzed by ICP for each sample. **(A).** The levels of P, K, Na, Ca, and Mg in shoots and roots of plants treated for two weeks. **(B).** The levels of Fe, Zn, Cu, Al, and Mn in shoots and roots of plants treated for two weeks. Data represents means from four independent biological replicates. Error bars indicate $\pm$ SE. No significant differences at $p < 0.05$ were observed among the treatments by Duncan's multiple range test. The other elements examined were at very low levels or absence and not shown here.

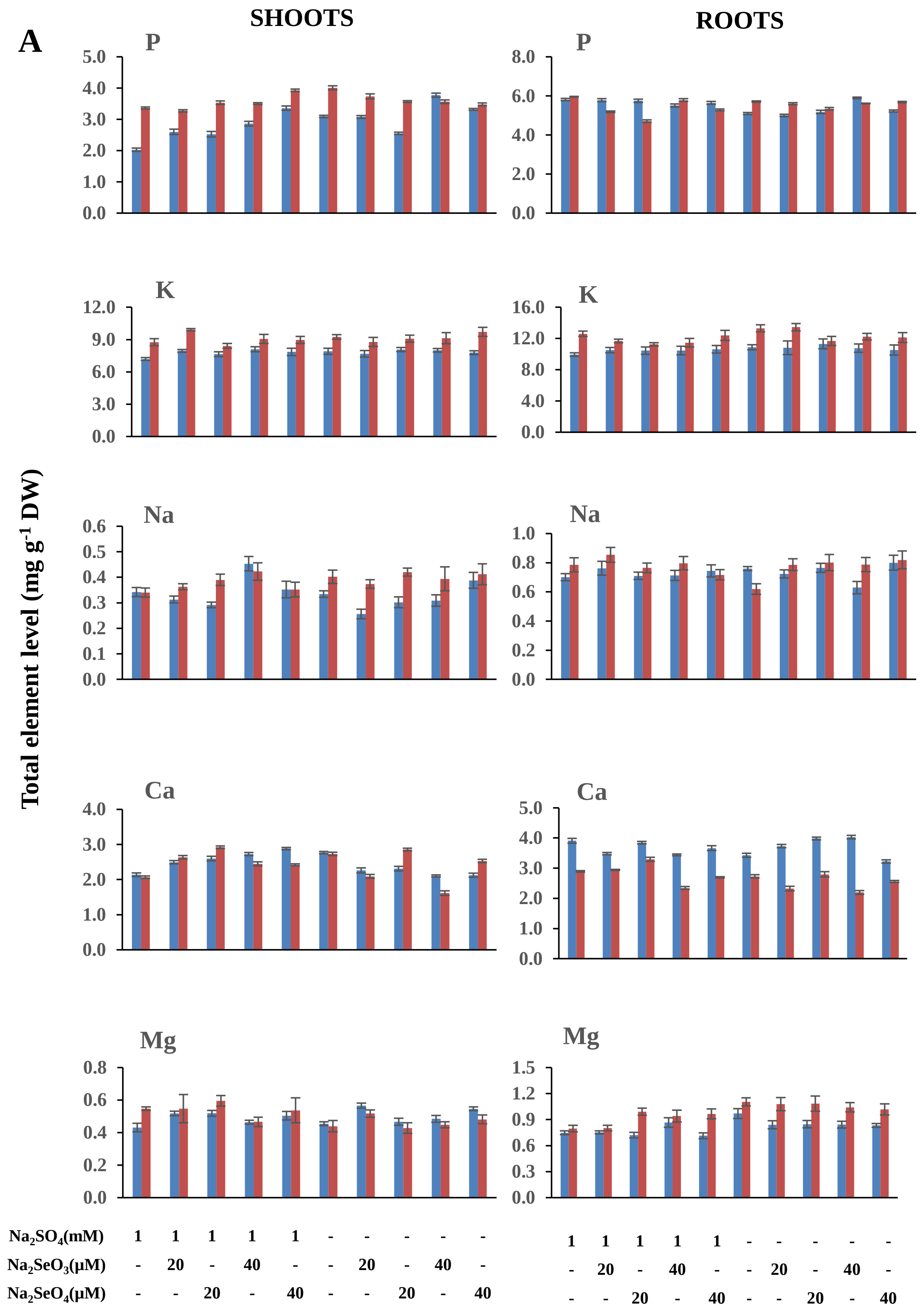

A
SHOOTS
ROOTS
P
K
Na
Ca
Mg
Total element level (mg g-1 DW)
Na2SO4(mM) 1 1 1 1 1 - - - - -
Na2SeO3(µM) - 20 - 40 - - 20 - 40 -
Na2SeO4(µM) - - 20 - 40 - - 20 - 40

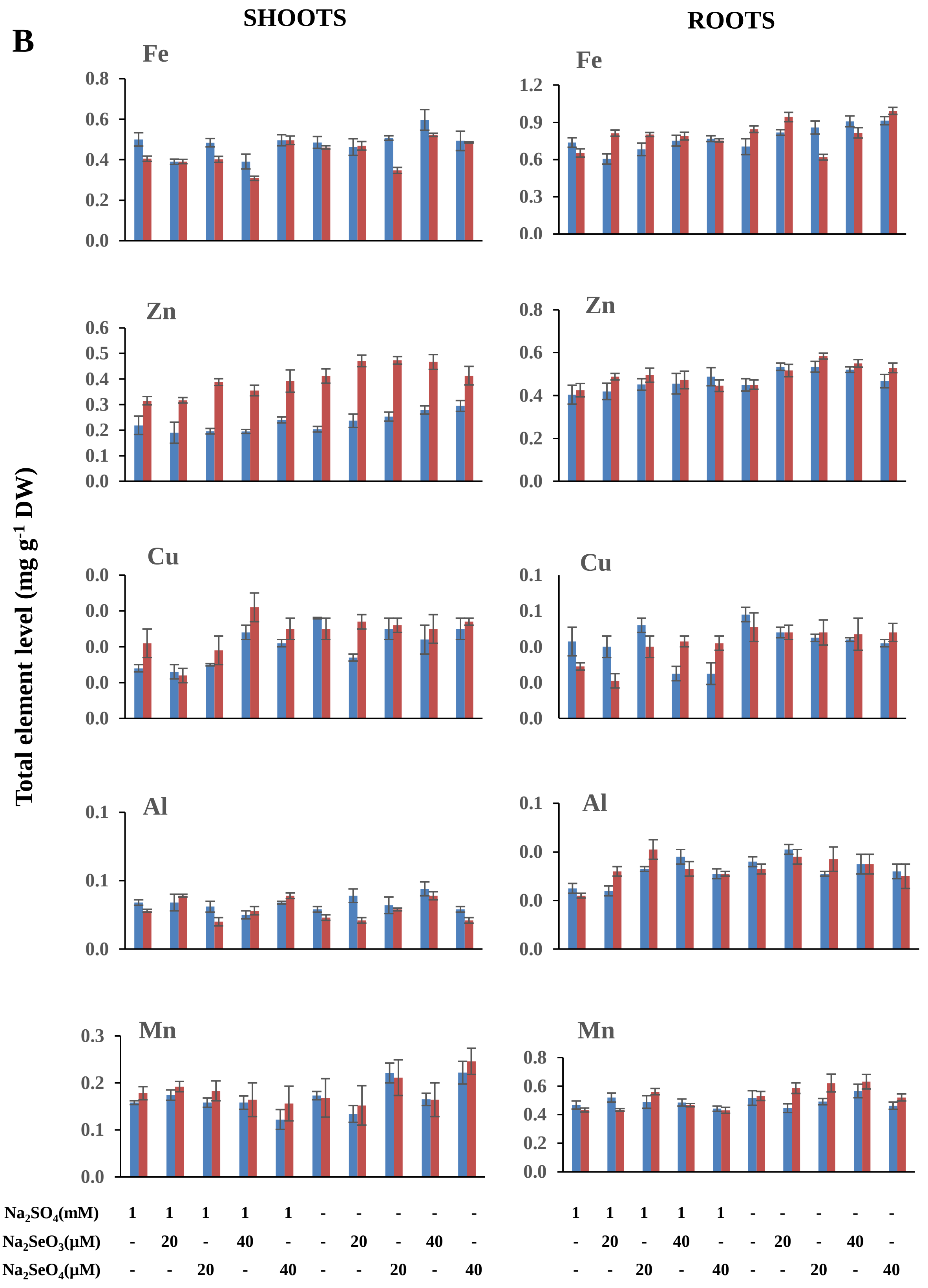

B
SHOOTS
ROOTS
Fe
Zn
Cu
Al
Mn
Total element level (mg g-1 DW)
Na2SO4(mM) 1 1 1 1 1 - - - - -
Na2SeO3(μM) - 20 - 40 - - 20 - 40 -
Na2SeO4(μM) - - 20 - 40 - - 20 - 40
